# “Cartilage Injuries of the Foot: What We Do Not Know, We Fear . . .”

**DOI:** 10.1177/19476035231216088

**Published:** 2023-12-09

**Authors:** Jari Dahmen, Masato Takao, Mats Brittberg, Gino M.M.J. Kerkhoffs

Dear Colleagues,

We extend a warm welcome to the inaugural special issue of the *CARTILAGE* journal focused on cartilage injuries of the foot. Cartilage injuries of the foot are considered rare entities with limited evidence available in the orthopedic scientific literature—the majority of the publications on cartilage lesions of the foot consist of case reports and retrospective research.^1-5^ When scrutinizing the literature on PubMed, only a handful of articles have been published primarily dedicated to the treatment of cartilage injuries of the foot. This paucity is mirrored by the limited number of patients with such injuries that we, as treating physicians, encounter annually. We can thus state that unfamiliarity breeds disregard, posing a clinical danger to patients suffering from these lesions as physicians may wrestle with insufficient knowledge to discern the most optimal course of treatment. Nevertheless, cartilage lesions of the foot are profoundly disabling, characterized by a diminished quality of life and a substantial disruption of daily functioning.^4,6,7^ This underscores the necessity of a more extensive and elaborate scientific understanding through the presently prepared Special Issue, emanating from diverse Centers of Excellence within the International Cartilage Regeneration & Joint Preservation Society (ICRS), aimed at delivering the soundest up-to-date evidence-based guidance to medical practitioners entrusted with the care of patients with cartilage lesions to the foot.

To kick off this special issue, Dr. Shimozono and his team performed a Systematic Review of clinical studies on osteochondral lesions (OCLs) of the subtalar, talonavicular, and calcaneocuboid joints.^8^ The authors confirmed their hypothesis that the currently available evidence is limited and that, as such, there is a specific and urgent need to publish an evidence-based treatment algorithm for OCLs of the abovementioned joints.

In addition to the overview of the current literature and in order to fill part of the research gap that was described by Dr. Shimozono *et al.*, New York University and Amsterdam UMC joined forces to shed new light on the clinical outcomes after treatment of (osteo)chondral lesions of the subtalar joint.^9^

Thereafter, various surgical techniques including clinical outcomes for the treatment of osteochondral lesions to the talonavicular joint were described and evaluated from the Boston and Amsterdam Perspectives which included a thorough overview of clinical pirls and pitfalls that can be easily applied in daily clinical practice.^10^

One stage further down the clinical cascade of focal damage is the presence of whole-joint isolated osteoarthritis. A fusion of the joint in question is considered an evidence-based procedure at the end of the decision-making process, and, as such, there is a clear wish and need for joint-sparing biological surgical procedures. Clinical and radiological outcomes of a novel biological resurfacing arthroplasty are presented using a Tensor Fascia Lata Autograft (BioJoint) for the surgical treatment of isolated osteoarthritis to one or more joints of the foot.^11^

Hereafter, this special issue continued by focusing on the surgical treatment outcomes of osteochondral lesions of the metatarsophalangeal-1 joint (MTP-1).^12^ Cartilage injuries of the MTP-1 joint are rare and warrant scientific attention to improve outcomes for the patient. As such, the clinical, work, and sports-related outcomes of surgical treatment of these injuries were studied, aiming to fill the grand research gap on these outcomes.

Yoshimura *et al*.^13^ then introduced a novel best available evidence-based treatment algorithm for Freiberg disease. The research project was led by Professor Yoshimura and included a multi-center international experts’ collaboration, ultimately resulting in new and updated insights on how to best treat a patient with Freiberg disease. This research article provides a clinical decision-making tool to assist decision-making for each individual stage of Freiberg disease.

The special issue is closed by Dr. Angthong and colleagues^14^ who set up a novel treatment algorithm consisting of an updated evidence-based summary on when to choose conservative management and which type of surgery for each specific stage of Muller-Weiss syndrome. This article supplies clinicians with a clinically relevant and applicable algorithm to aid with decisions for each stage of Muller-Weiss syndrome.

This special issue of the *CARTILAGE* journal is composed of research manuscripts that have aimed to ameliorate the evidence on cartilage injuries of the foot. We hope that this will help the reader to extend his or her journey in pursuing clinical excellence when treating these rare injuries and we would like to express our sincere gratitude to the editorial team and the authors who all put in their greatest efforts. Finally, we can state that the intensive and fruitful international collaborations allowed for upgrading the clinical evidence for our future patients who suffer from cartilage lesions of the foot.


Jari Dahmen Department of Orthopedic Surgery and Sports Medicine, Amsterdam University Medical Centers, Location Academic Medical Center, Amsterdam Movement Sciences, University of Amsterdam, Amsterdam, The Netherlands Amsterdam Collaboration for Health and Safety in Sports, Academic Medical Center/VUmc IOC Research Center, Amsterdam, the Netherlands
Masato Takao Clinical and Research Institute for Foot and Ankle Surgery, Jujo Hospital, Kisarazu, Japan
Mats Brittberg Cartilage Research Unit, Region Halland Orthopaedics, Kungsbacka Hospital, University of Gothenburg, Kungsbacka, Sweden
Gino M.M.J. Kerkhoffs Department of Orthopedic Surgery and Sports Medicine, Amsterdam University Medical Centers, Location Academic Medical Center, Amsterdam Movement Sciences, University of Amsterdam, Amsterdam, The Netherlands Amsterdam Collaboration for Health and Safety in Sports, Academic Medical Center/VUmc IOC Research Center, Amsterdam, the Netherlands

